# An electron microscopic study of the archaeal feast/famine regulatory protein: 4. Estimation of the particle size

**Published:** 2004-03-01

**Authors:** Lester Clowney, Sanae A. Ishijima, Masashi Suzuki

**Affiliations:** *) National Institute of Advanced Industrial Science and Technology (AIST), AIST Tsukuba Center 6-10, 1-1-1, Higashi, Tsukuba, Ibaraki 305-8566, Japan; **) Japan Science and Technology Agency (JST), Core Research for Evolutionary Science and Technology (CREST), Honmachi, 4-1-18, Kawaguchi Center Building, Kawaguchi, Saitama 332-0012, Japan

**Keywords:** Contrast transfer function, cryo-electron microscopy, Fourier analysis, hyper-thermophile, image processing

## Abstract

Using the method of Fourier transform, cryo-electron micrographs of two types of archaeal feast/famine regulatory proteins (FFRPs), pot0434017 (FL11) and pot1216151 (DM1), were analyzed. After correcting the Fourier power spectra by considering effects of the contrast transfer functions (CTFs), peaks were identified at frequencies, corresponding to the particle size of ~130 Å for FL11 in the complex with DNA, in addition, a smaller size, ~40 Å, for the same protein in the absence of DNA, the particle size of ~65 Å for DM1 when interacting with a ligand, and a smaller size of ~30 Å when the ligand was removed. These numbers are consistent with our previous observations that dimers of FL11 form octamers, i.e. tetrameric assemblies of the dimers, upon intercation with DNA, and that similar octamers of a smaller FFRP, DM1 of the molecular weight approximately half that of FL11, are stabilized by interaction with the ligand. Some aspects of CTF correction are discussed.

## Introduction

We have been studying the structure and function of a group of bacterial transcription factors, the feast/famine regulatory proteins (FFRPs).1)–10) These proteins have multiple assembly forms, and alternation between such assembly forms is important for transcription regulation of genes by FFRPs. In this study, two types of FFRPs in different assembly forms were studied using cryo-electron microscopy: pot0434017 (FL11) and pot1216151 (DM1) from the hyper-thermophilic archaeon *Pyrococcus* sp. OT3. The molecular weight of FL11 is about twice that of DM1. Namely, DM1 is composed of a single (i.e. assembling) domain, while in FL11 a DNA-binding domain is linked to the assembling domain, which corresponds to the whole DM1.

In order to estimate the overall diameters of assemblies of the two FFRPs (i.e. the particle sizes), their EM images were analyzed by the method of Fourier transform. When protein molecules are not crystallized, in a Fourier transform of the electron micrograph the size information is given by rings formed at particular spatial frequencies: in what follows, each 2D Fourier power spectrum (FPS) is radially averaged, yielding a 1D plot. Here the problem is that such a plot is not free from effects of distortion caused in the process of electron optical imaging. In short, “seeing” can be misleading, and efforts to minimize such effects are necessary.11)

Biomolecular assemblies scatter or absorb electrons only weakly, and thus no contrast is expected when the image is in focus: these objects are as “invisible” as a piece of glass in water. Shifting of the phase of scattered electrons relative to that of the unscattered electrons, is the only way to obtain contrast, i.e. phase contrasting.11)–13) In optical microscopy this is achieved using a physical device (i.e. the Zernike phase plate), but in electron microscopy it is usually done by changing an operational condition, i.e. underfocussing of the objective lens.

Upon defocusing, phases are not all changed by the same amount. The spherical aberration of the objective lens and the defocus combine to affect the image by enhancing or weakening the contrast depending on the spatial frequency. Between a pair of frequencies at which the contrast is enhanced, at an intermediate frequency the contrast becomes zero. The function describing this oscillation is called the contrast transfer function (CTF). As will be discussed in this paper, in order to determine the particle size, identification of the first peak, or more precisely its high frequency end in the FPS is needed, and for this, effects of CTF needs to be corrected by deconvolution.

## Preparation of FFRPs in various forms

The protein FL11 was expressed and purified as has been described,2),8) and its complex with DNA was made.2) This DNA has 91 basepairs, and wraps around the octameric assembly of the protein: the DNA complex of FL11 (C), distinguished from the DNA-free form (F).

The protein DM1 was expressed, purified, and prepared in two forms as has been described.4),14) In one of these forms, here referred to as the assembled (A) form, the protein is stabilized into octamers, similar to those of FL11, by interaction with a ligand present in *E. coli* cells. In the other form, here referred to as the disassembled (D) form, the ligand is removed and the octamers are disassembled into smaller assemblies.

## Cryo-electron microscopy

A droplet, 4 μl, of each protein solution, 40 μg/ml in 50 mM Tris-HCl buffer (pH = 7.0) containing 200 mM NaCl, was placed on a holey carbon-coated copper grid (300 mesh, Electron Microscopy Sciences Co.). In order to enhance the scattering, the proteins were stained using a heavy metal, i.e. 16% ammonium molybdate, by the method of Adrian *et al*.15) As was expected, the protein DM1 was negatively stained in both forms, A and D. While, FL11 (F) and its DNA complex (C) were positively stained, possibly due to the staining time of ~90 sec, which is longer than that used for DM1, 30 sec or less.

As before,2)–5) the specimen was kept in amorphous ice in order to minimize damage caused by dehydration and electron irradiation, i.e. the method of cryo-electron microscopy.16) The grid was quickly frozen in liquid ethane into an amorphous ice state, using a freezing apparatus (EM CPC, Leica). The grid was maintained at a near liquid nitrogen temperature using a holder (CT3500, Oxford), while an electron microscope (Tecnai F20, FEI) was operated at 200 KeV. The spherical aberration coefficient (C*s*) of the objective lens of this microscope is 2.0 mm.

**Fig. 1:**
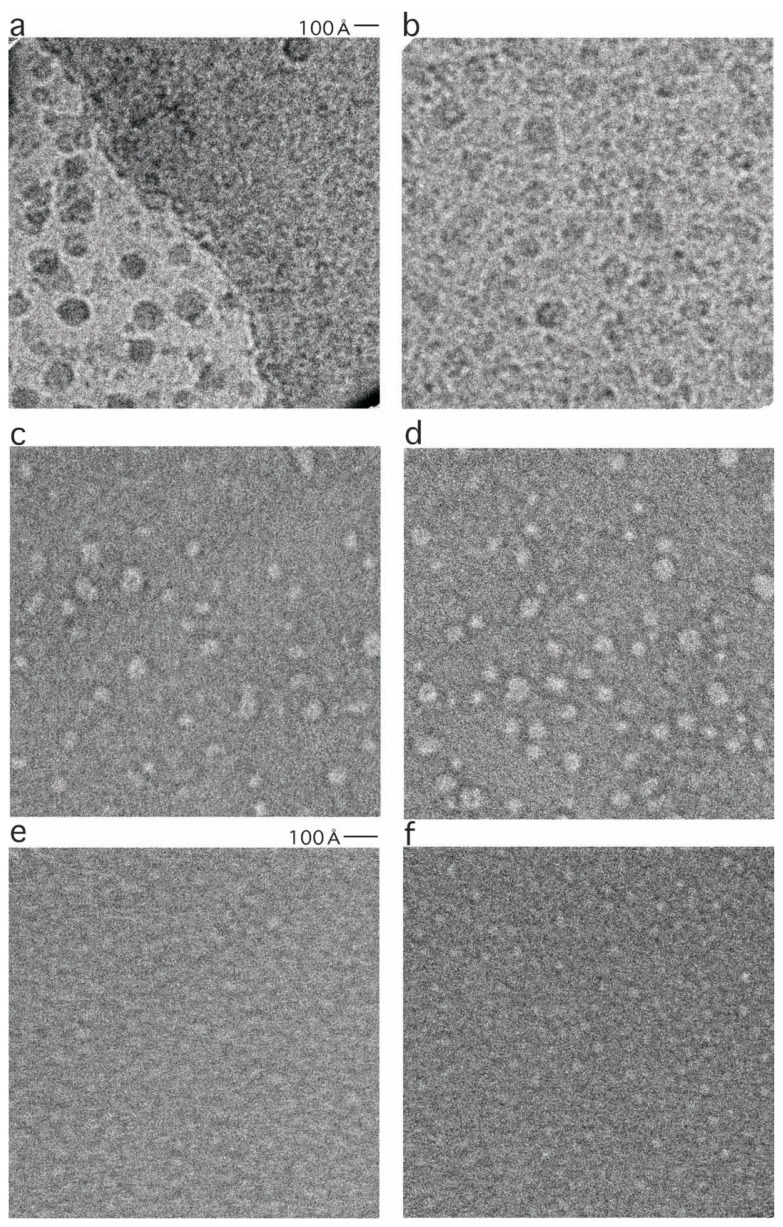
Electron micrographs of FL11 in the complex with DNA (a, FL11C15), FL11 in the DNA-free form (b, FL11F09), and DM1 in the assembled (c, DM1A4 and d, DM1A5) or disassembled (e, DM1D02 and f, DM1D10) forms. These show the whole area covered by CCD. Note that the magnification of a and b is different from that of c–f; note the two scale bars.

To minimize chromatic aberration, electrons that had lost no energy (i.e. 200 KeV±10 eV) were selectively focused using an energy filter (GIF200, Gatan), and recorded using a CCD camera (794IF, Gatan, 1,024 pixels × 1,024 pixels) and Digital Micrograph software (Gatan). The magnification of the CCD images was 155 K (FL11) or 230 K (DM1), with each pixel corresponding to 1.54 Å (FL11) or 1.04 Å (DM1). Examples of the electron micrographs obtained are shown in Fig. 1.

## Fourier transform and CTF correction

**Fig. 2:**
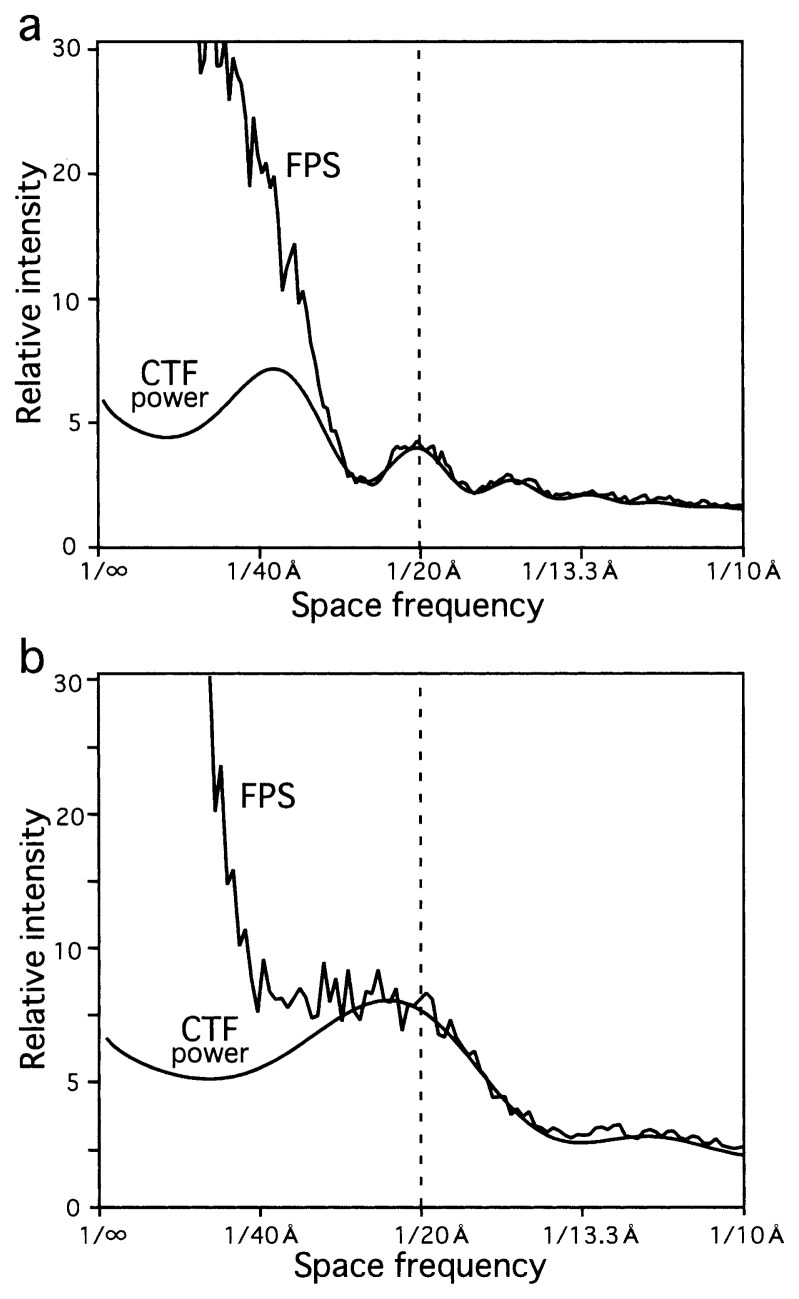
Examples of CTF fitting to FPS of electron micrographs (a, DM1A3 and b, FL11C17). The whole area covered by CCD was used for calculating the FPS. Aiming at best fitting, high frequency regions of FPS were included upon the fitting (the right halves). For the estimation of the particle size, only frequencies lower than 1/20 Å (indicated) were needed (see Figs. 4 and 5).

Using the Eman package,17) Fourier power spectra (FPS) of the four sets of images (Table I) were calculated, and radially averaged (Fig. 2). By using the same package,17),18) the CTF correction of the FPS was carried out.

**Fig. 3:**
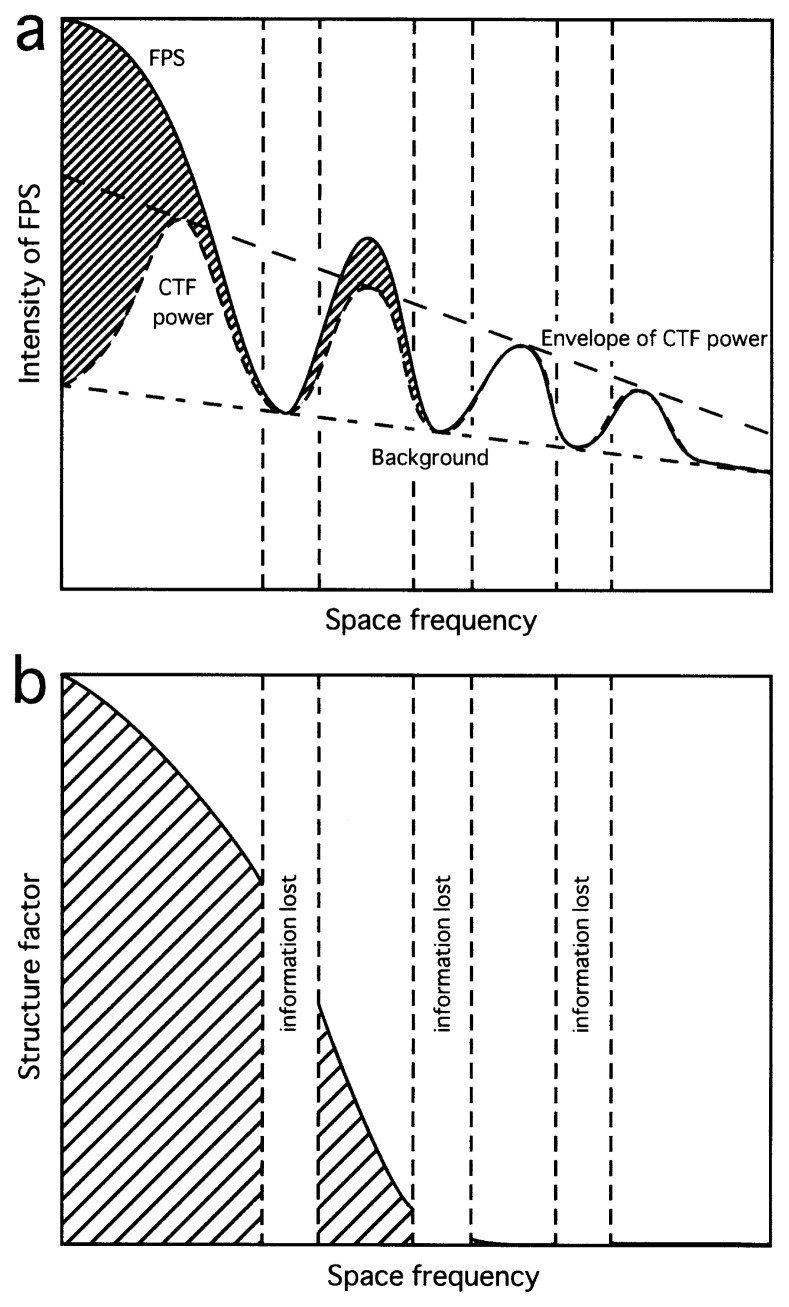
A schematic drawing showing CTF fitting to the Fourier power spectrum (FPS) (a), and another drawing showing the structure factor calculated by deconvolution of FPS with CTF (b).

First, for each FPS the background was defined (Fig. 3a), so that at high frequencies it would match with the original FPS, and at lower frequencies it should match with local minima periodically occuring: these minima are reflections of the CTF, and at these frequencies the CTF and therefore the FPS, where the CTF is convoluted, should have no intensity.

Then, a curve showing the power of CTF, was fitted to the background-free FPS, so that the CTF power would not exceed the intensity of FPS at any frequency, beyond the noise level (see examples in Fig. 2). Here, the corrected FPS, i.e. the background-free FPS divided by the CTF power, defines the corrected structure information, i.e. the structure factor (Fig. 3b). Values of the structure factor are larger than 1 by definition.

For this CTF fitting, three types of parameters were adjusted. One of the three defines the oscillation of the fitted CTF, and this parameter alone defines the degree of defocusing (Table I), with a higher oscillation associated with a larger defocus value. While, the other two parameters define the overall shape (i.e. the envelope) of the CTF power plot: the intensity at the zero frequency and the rate of decay towards higher frequencies. The spatial resolution which can be achieved by an electron microscope has a limitation, producing a decay (or damping) in the FPS towards the high frequency end.

The oscillation of FPS, originating in that of CTF, might be removed by averaging FPS of a large number of electron micrographs taken with different defocus values. However, the inclination caused by the Fourier decay or the background cannot be removed by the simple averaging. The essence of the CTF correction is to define CTF by the oscillation of FPS, and by using the identified CTF to define the background and the damp, thereby calculating the structure factor.

## Recovering of the missing information

**Fig. 4:**
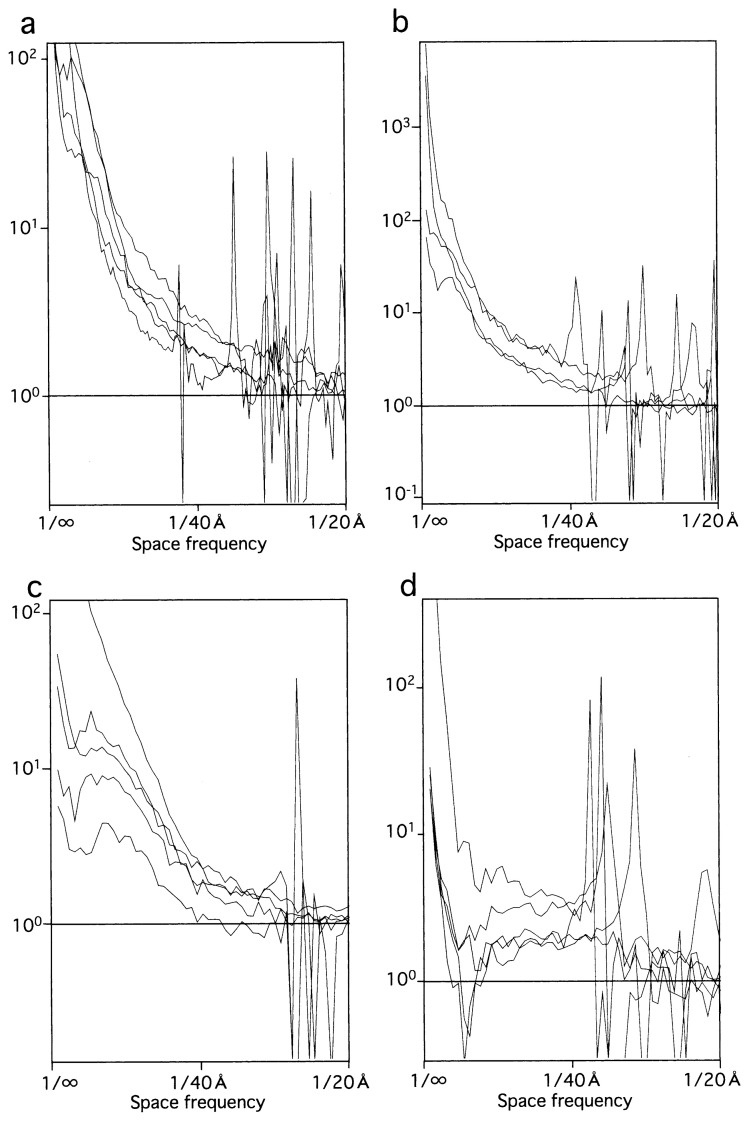
Sets of structure factors calculated for C, i.e. FL11 in the complex with DNA, (a), F, i.e. FL11 in the DNA-free form, (b), A, i.e. DM1 in the assembled form, (c), and D, i.e. DM1 in the disassembled form, (d).

One might expect that after the CTF correction, all spatial frequencies could contribute to the contrast with the same magnitude. However, between a pair of peaks in the CTF power, or more precisely, between negative and positive peaks in the original CTF, at a particular frequency the value of the CTF becomes zero (Fig. 3a). At that frequency, the structure factor is not defined (Fig. 3b): the information is lost at such a frequency. In the plots of structure factors calculated using the four sets of electron micrographs (Fig. 4), such points have been removed. However, still sharp spikes are turning up around these frequencies, reflecting the loss of information.

In some cases such information loss might not be important. For the A set (Fig. 4c), the spikes are found at high frequencies only, and real peaks of the structure factors present at lower frequencies can be identified. However, for e.g. the D set (Fig. 4d), spikes overlap real peaks. In order to observe the poorly contrasted objects in D (compare Fig. 1e and f with c and d), higher defocusing was required, but this has lowered the first zero frequencies of the CTFs, thereby masking the structure factors in the region most important for observing the small particles.

**Fig. 5:**
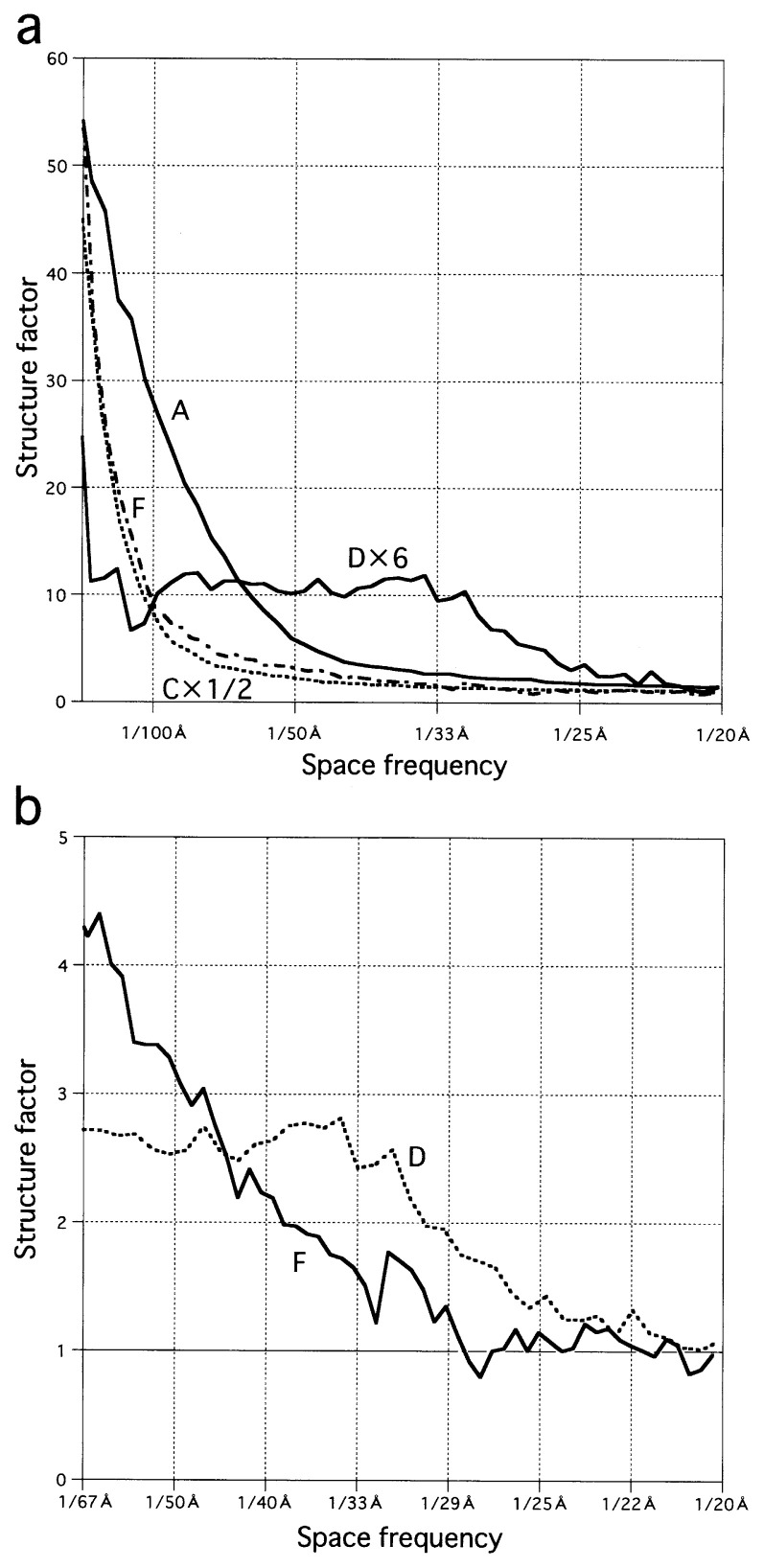
Structure factors averaged inside each set. In (a) for a better comparison the intensity of C is decreased by a factor of 2, and that of D is increased by a factor of 6. In (b) sections of D and F are enlarged. Note that the structure factor should not decrease below 1 by definition.

Electron micrographs are usually taken with different degrees of defocusing (Table I). By using such a series, it is possible to complete the information by filling the missing frequencies with each other. Accordingly, from each plot in the sets C, D and A, spikes were manually deleted, and the gaps were filled with the average values of the two frequencies each presenting before and after the gaps. This simple processing, together with averaging of 4–5 plots in each set, performed considerable improvements (compare Fig. 4 with Fig. 5a).

For the F set, more careful treatment was necessary. A similar gap filling was achieved for the frequencies up to 0.02 (i.e. 50 Å) and over 0.039 (i.e. ~25 Å). Between these frequencies, many spikes were found in all of the five plots, and similar gap filling was not effective. These spikes were removed and left unfilled, and the values observed in the other curves were averaged at the frequencies, after normalizing the five plots using an equation: [the corrected intensity at a frequency] = [(the intensity observed at the frequency)-a]/(b-a) × 2.08 + 1, where a and b were the intensities observed at 0.02 and 0.039, respectively, in each plot. With this treatment, all the four plots were normalized to have values, 3.08 at 0.02 and 1.00 at 0.039, the average values of the four original plots at the two frequencies.

## Particle sizes

The average structure factors calculated for the four sets, DM1 disassembled (D), DM1 assembled (A), FL11 DNA-free (F) and FL11-DNA complex (C), have some notable differences, reflecting differences in the particle sizes (Fig. 5a). Larger distance information is present at lower spatial frequencies in the Fourier space: the presence of peaks at the lowest frequencies in C and F indicates formation of the largest particles. The curvatures of F and C are similar, but the intensity of C is approximately twice that of F: assemblies of similar sizes are formed, but the number of such assemblies in C is twice that in F. The plot of A extends to higher frequencies, suggesting formation of smaller assemblies, and that of D extends even further suggesting that of the smallest particles.

**Fig. 6:**
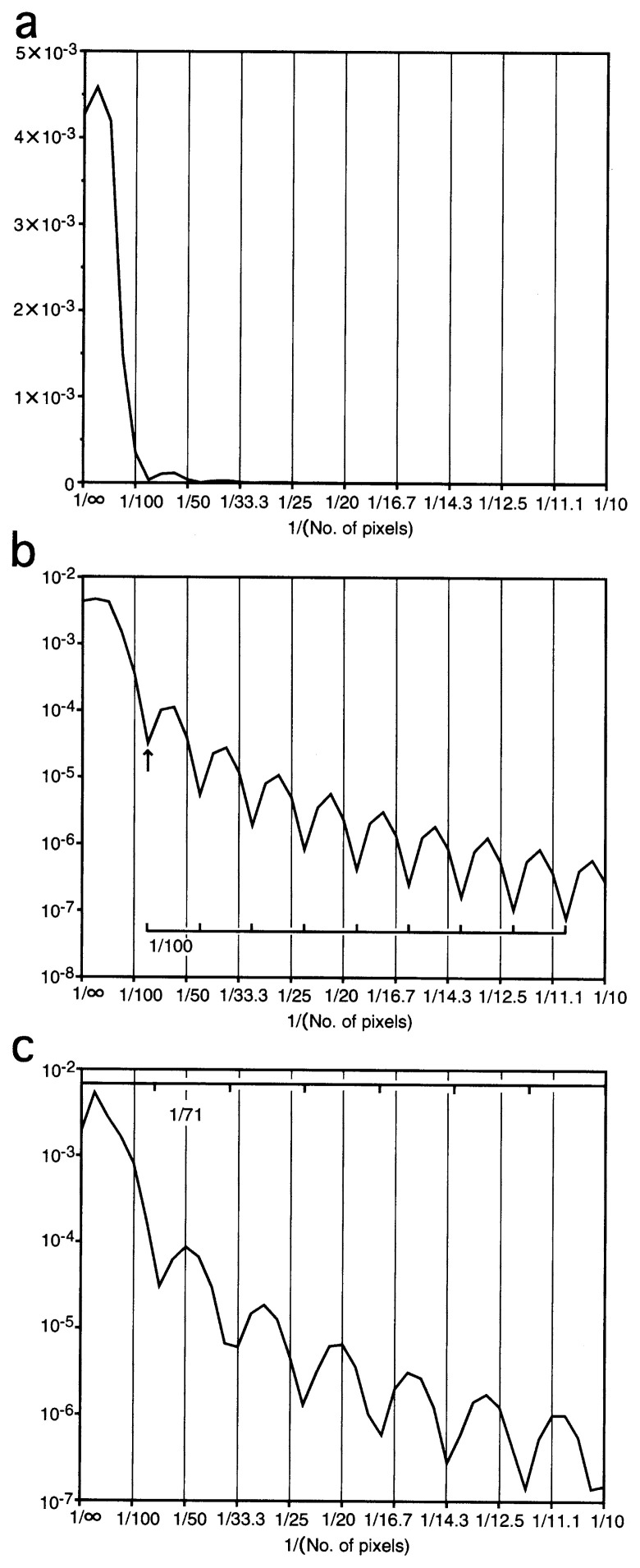
Fourier power spectra (FPS) of circles of the diameter of 100 pixels (a, b) and squares of the diagonal lengths of 100 pixels (c), randomly distributing in the plane 1,024 pixels × 1,024 pixels. In (a) the y axis is expressed in a linear form, but in (b) and (c) the y axes are expressed in logarithmic forms. In (b) the first minimum is observed at around 1/80 pixels (indicated by an arrow), a value close to 1.22 × [1/100], which is followed by a series of minima with a periodicity of 1/100 (highlighted by a scale). In (c) a series of minima are observed with a periodicity of 1.41 × [1/100] through the plot.

The Fourier transform of a circular object is described by using the Bessel function. In short, the first minimum is expected at the space frequency 1.22/*d*, where *d* is the diameter (Fig. 6a, b). If the object is not totally circular but, in fact, closer to a square, as is the case for the A form of DM1,4) the minimum is found at 1.41/*d*, where *d* is the diagonal length (Fig. 6c). For a perfect circle or a square, series of minima will follow with a periodicity of 1/*d* (Fig. 6b) or 1.41/*d* (Fig. 6c). However, in any case, peaks decrease dramatically (Fig. 6a, note that the y axes in Fig. 6b, c are expressed in logarithmic forms). It is very difficult to observe such small peaks, because of the resolution limit of the electron microscope.

Using the first minima, i.e. the high frequency ends of the first peaks in the structure factors, now it is possible to conclude that the particle sizes of the large assemblies in C and F will not be so different from 130 Å (Fig. 5a, Table I). That of DM1 in A will be ~65 Å, and that in D ~30 Å (Fig. 5b, Table I). When looked at more carefully, the two curves of F and C are not totally the same, that of F being higher at around 50 Å (Fig. 5a). In fact, F has another component, suggesting the presence of particles of ~40 Å (Table I). Small particles of such sizes are visible in the original electron micrographs (Fig. 1b).

The above observations are consistent with our previous conclusions.2),4) Namely, dimers of FL11 further assemble into octamers, i.e. tetrameric assemblies of the dimers, upon intercation with DNA. Similar octamers of DM1 are stabilized by interaction with the *E. coli* ligand. Thus, when the two forms of each protein are compared, the estimated particle sizes differ by a factor of 2.2–3.3. While in the same assembly forms, octermeric or dimeric, the sizes of the two proteins differ by a factor of 1.3–2.0. These numbers are not so different from what are expected from the 2 (FL11) to 1 (DM1) ratio of the molecular weights and the crystal structures of the two proteins.

## Figures and Tables

**Table I:** Statistics of the electron micrographs analyzed

FL11 DNA complex	(C form)	

ID	Magnification	Defocus
FL11C15(LrpDNA-15-2)	155 K	**2.9** μm
FL11C16(LrpDNA-16)	155 K	**3.3** μm
FL11C17(LrpDNA-17-1s)	155 K	**2.4** μm
FL11C18(LrpDNA-18-2)	155 K	**2.8** μm
FL11C19(LrpDNA-19)	155 K	**2.2** μm

	1st Minimum	1st Min. × (1.22–1.41)

	100 Å	**122–141 Å**

FL11 DNA free form	(F form)	

ID	Magnification	Defocus
FL11F09(Lrp-09)	155 K	**4.3** μm
FL11F10(Lrp-10)	155 K	**3.2** μm
FL11F11(Lrp-11)	155 K	**3.2** μm
FL11F12(Lrp-12)	155 K	**5.6** μm

	1st Minimum	1st Min. × (1.22–1.41)

	100 Å	**122–141 Å**
	30 Å	**37–42 Å**

DM1 Assembled form	(A form)	

ID	Magnification	Defocus
DM1A1(S01-06-200K-15)	230 K	**4.9** μm
DM1A2(S01-06-200K-07-16)	230 K	**4.7** μm
DM1A3(S01-06-200K-14)	230 K	**4.7** μm
DM1A4(S01-06-2-200K)	230 K	**2.1** μm
DM1A5(S01-06-3-200K)	230 K	**2.2** μm

	1st Minimum	1st Min. × (1.22–1.41)

	50 Å	**61–71 Å**

DM1 Disassembled form	(D form)	

ID	Magnification	Defocus
DM1D02(S01-02-20002)	230 K	**4.4** μm
DM1D10(S01-02-20010)	230 K	**2.6** μm
DM1D11(S01-02-20011)	230 K	**3.7** μm
DM1D12(S01-02-20012)	230 K	**3.4** μm
DM1D13(S01-02-20013)	230 K	**2.3** μm

	1st Minimum	1st Min. × (1.22–1.41)

	22 Å	**27–31 Å**
